# Risk factors of renal dysfunction and their interaction in level-low lead exposure paint workers

**DOI:** 10.1186/s12889-018-5475-9

**Published:** 2018-04-20

**Authors:** Xiaojuan Wang, Huiling Liang, Yan Wang, Chang Cai, Jimeng Li, Xun Li, Mian Wang, Mengshi Chen, Xin Xu, Hongzhuan Tan

**Affiliations:** 1 Department of Epidemiology and Health Statistics, School of Public Health, Central South University, RD Xiangya 90, Changsha, Hunan 410008 People’s Republic of China; 2 Changsha Center for Diseases Control and Prevention, Changsha, Hunan 410001 People’s Republic of China; 3 National Center for AID/STD Control and Prevention, Chinese Center for Diseases Control and Prevention, Beijing, 102206 People’s Republic of China

**Keywords:** Risk factors, Renal insufficiency, Lead, Hypertension

## Abstract

**Background:**

To explore the effect of low-level lead exposure on renal dysfunction in paint works, and analyze the interaction between low-level lead exposure and other influence factors of renal dysfunction.

**Methods:**

Seven hundred forty seven workers from Sany Heavy Industry Company and Xiangjiang Kansai Paint Company who have been exposed to paint were chosen by random cluster sampling. Their blood lead level and Urine β2-micro globulin level (renal dysfunction) were tested,risk factors of renal dysfunction in paint workers and their interactions were analyzed.

**Results:**

The prevalence of renal dysfunction was 12.37%. Risk factors of renal dysfunction in paint workers mainly included longer working years (OR = 1.699, 95% CI: 1.226~ 2.355), blood lead positive (OR = 2.847, 95% CI: 1.577~ 5.139) and hypertension (OR = 2.192, 95% CI: 1.103~ 4.359). Positive interaction existed between hypertension and low-level blood lead on renal dysfunction in paint workers, the RERI (Relative excess risk of interaction), API (Attributable proportions of interaction) and S(the synergy index) were 4.758, 54.5% and 2.604 respectively.

**Conclusions:**

Low-level lead exposure and hypertension not only have independent effect on renal dysfunction in paint workers, but also had obvious positive interaction in paint workers. Interventions aimed at blood lead and blood pressure at the same time will better prenvent from renal dysfunction.

**Electronic supplementary material:**

The online version of this article (10.1186/s12889-018-5475-9) contains supplementary material, which is available to authorized users.

## Background

Chronic lead poisoning may cause malfunction of a variety of organ systems. Since Lanceraux E [1] reported the nephrotoxic effects of lead in 1881, a great deal of researches have been done on renal dysfunction caused by exposure to high level of lead [2, 3]. With further understanding of the lead, government has tightened the supervision of enterprises and enterprises also have raised the awareness of keeping the lead level in workplace within limit. Therefore, the level of lead in the related occupational environments has been reduced by a large margin. Recently, a large number of researches have been devoted to studying the damage of low level lead exposure to the kidney. However, results are inconsistent. Some found that low level lead exposure would result in nephrotoxicity [4, 5], while some large population studies showed that low level blood lead causes no harm to the kidney in general people [6]. It’s worth noting that in most of those studies, serum creatinine has been used as index of renal dysfunction and this might be a limitation since the level of serum creatinine is easily influenced by some other factors like examinees’ diet and metabolism, which could become abnormal at the terminal stage. In addition, most of these studies only focused on differences of serum creatinine between renal dysfunction and renal normal group but neglected other possible effects such as the interaction between blood lead and other factors.

Taking urine β2- microglobulin as index of renal dysfunction and taking workers in either paint using or painting producing enterprises with low level lead exposure as study populations, this paper studied the association between low level of lead exposure and the renal dysfunction and investigated the interactions between low level lead exposure and other factors. The findings could serve as the reference evidence for the formulation of relevant laws and regulations on prevention and control the harm of low level lead exposure.

## Methods

### Study population

According to the size of the factory, random cluster sampling was adopted in this investigation. There are 78 enterprises that either produce paint or use paint in Changsha, Hunan, China, whose harmful substance has been detected qualified. Finally, two enterprises namely Sany Heavy Idustry and Xiangjiang Kansai Paint Company, which are paint maker and paint user respectively, were chosen randomly. All workers exposed to paint in both companies during 2012 were chosen as the study population. The investigation was carried out from Oct. 2012 to Dec. 2012.

### Investigation

The purpose and process of this research were carefully explained to the study population, then, the written informed consent was obtained from all participants. Afterwards, all participants were required to answer the designed questionnaire and accomplish laboratory testing.

### Questionnaire survey

Questionnaire (Additional file 1: Questionnaire) designed by the research group were given to the paint workers before physical examination. Participants were asked to fill in the questionnaires under the guidance of investigators who have received uniform training face to face. The major information included in the questionnaires was age, average annual income in whole family, education, working year, whether smoke or nor, whether drink or not, whether wear masks on work or not, whether wash hands before meals or not, and so on.

### Laboratory testing

For lead quantification, 3 ml whole blood was collected by anticoagulant tubes from all participants before breakfast in the morning. The laboratory assessment of renalfunction and other parameters was done in Changsha Center for Diseases Control and Prevention (CDC) for Environmental Health following standardized protocols, collection and storage materials were not contaminated with background lead. Graphite furnace atomic absorption spectrophotometry (WS/T20–1996) was used for the detection of blood lead, the atomic absorption spectrophotometer is Thermo M6 manufactured by Thermo Electron Corporation, the internal controls are used for quality control and the detection limit of blood lead is 0.04 mg/l. For urine β2-MG testing, random urine was collected by clean and dry disposable urine cups and Sinnovwa D-360 fully–automatic biochemistry analyzer was utilized to detect the urine β2-MG based on particle enhanced immunoturbidimetric method.

### Physical examination

Physical examination was performed by medical practitioners from Changsha CDC. Blood pressure, height, weight and heart rate are included in the examination. Blood pressure was measured by mercury sphygmomanometer with cuff compression method with sitting and quiet conditions. The blood pressure of each subject was measured for three times and the mean value was available.

### Definition of indicators

Renal dysfunction: urine β2-MG ≥ 0.3 mg/L as the index of early renal dysfunction [7]; Blood lead positive: Pb-B ≥ 0.04 mg/l which is the detection threshold of above testing method; Blood lead negative: Pb-B < 0.04 mg/l; Low-normal blood pressure: systolic pressure < 120 mmHg and diastolic pressure<80 mmHg; High-normal blood pressure: 120 mmHg ≤ systolic pressure < 140 mmHg or 80 mmHg ≤ diastolic pressure < 90 mmHg; Hypertension: systolic ≥ 140 mmHg or diastolic pressure ≥ 90 mmHg; Smoking: one cigarette or more in one month; Alcohol drinking: once a week on average and with a drinking history of 6 months or above. Individual health protection such as wearing mask and washing hands before meals et al. less than once was regarded as never, more than or equal to once but less than or equal to three times was regarded as occasionally while more than three time a week was considered as frequently.

### Statistical analysis

Epidata 3.0 was adopted to establish a database. SPSS 19.0 was used to analyze the data. The continuous variables like age and working year were divided into three grades according to its percentiles of 25 and 75. Variables like smoking, drinking, wearing mask, blood lead and blood pressure were divided into two or more categories according to above definitions. Chi-square test and multi-factor logistic regression analysis was used to analyze risk factors of renal dysfunction. The interaction between the blood lead and blood pressure was analyzed by stratified analysis. Based on the OR values of every stratification, RERI (relative excess risk of interaction), API (attributable proportions of interaction) and S (the synergy index) were calculated to evaluate the interaction. The statistical significance level was set at α = 0.05. All statistical tests were 2-tailed (Table 1).

**Table 1:** Variable assignment of unconditional logistic regression analysis of risk factors of renal dysfunction in paint workers

Variable	Definition and variable assignment
Renal dysfunction	No = 0, Yes = 1
Age (years)	< 25 = 1, 25~ = 2, 37~ 59 = 3
Gender	Male = 1, Female = 2
Work year (years)	< 2 = 1, 2~ = 2, 9~ 32 = 3
Smoke	No = 0, Yes = 1
Drink	No = 0, Yes = 1
Wear mask	Never = 1, Occasionally = 2, Frequently = 3
Wash hands before meals	Never = 1, Occasionally = 2, Frequently = 3
Blood pressure	Normal blood pressure = 0, Hypertension =1
Blood lead	Negative = 0, Positive = 1

### Measures of interaction on an additive scale

For two dichotomous factors A and B: RR_A + B+_ is the relative risk of disease if both factors A and B are present, RR_A + B-_ is the relative risk of disease if factor A is present but factor B is absent, RR_A-B+_ is the relative risk of disease if factor A is absent but factor B is present. Moreover, with logistic regression, one needs the assumption that the odds ratio approximates relative risk to remove the dependence on the covariates of the test and of the measures of departure from additivity.

1. Relative excess risk due to interaction (part of the total effect that is due to interaction):

$$ \mathrm{RERI}={\mathrm{RR}}_{\mathrm{A}+\mathrm{B}+}\hbox{-} {\mathrm{RR}}_{\mathrm{A}+\mathrm{B}\hbox{-}}\hbox{-} {\mathrm{RR}}_{\mathrm{A}\hbox{-} \mathrm{B}+}+\kern0.37em 1 $$

RERI = 0 means no interaction or exactly additivity; RERI > 0 means positive interaction or more than additivity; RERI<0 means negative interaction or less than additivity; RERI can go from - infinity to + infinity.

2. Proportion attributable to interaction (proportion of the combined effect that is due to interaction):

$$ \mathrm{AP}=\frac{RERI}{RR_{A+B+}} $$

AP = 0 means no interaction or exactly additivity; AP>0 means positive interaction or more than additivity; AP<0 means negative interaction or less than additivity; AP can go from − 1 to + 1.

3. Synergy index (ratio between combined effect and individual effects):

$$ \mathrm{S}=\frac{RR_{A+B+}-1}{\ \left(\ {RR}_{A+B-}-1\ \right)+\left({RR}_{A-B+}-1\ \right)} $$

S = 1 means no interaction or exactly additivity; S > 1 means positive interaction or more than additivity; S < 1 means negative interaction or less than additivity; S can go from 0 to infinity.

## Results

A total of 780 workers in the two chosen companies met the standard of exposing to paint for at least one year and 752 of them participated in this investigation, yield a 96.41% response rate. Of the 752 participants, 747 has valid result for urine β2-MG, making the valid survey rate as high as 98.13%. Of the 747 workers, 348 were from Sany Heavy Industry and 399 were from Xiangjiang Kansai Paint Company. The participants’ age ranged from 18 to 59 years old (mean age 31.66 ± 7.74 years old). The study population was made up of 706 males and 41 females. Of the study population, 70 workers were blood lead positive, accounting for 9.37% in total participants. The blood lead level in blood lead positive workers was between 0.04 and 0.35 mg/l (mean 0.09 ± 0.06 mg/l), which was below the limit of blood lead level stipulated by Chinese government for the relevant occupations (of 0.40 mg/l). Urine β2- MG level was between 0.02 and 0.69 mg/l (mean 0.20 ± 0.10 mg/l) in all subjects. 93 workers were diagnosed as renal dysfunction, which yield the renal dysfunction rate as 12.37%.

The results of univariate analysis showed that renal dysfunction prevalence rate were higher among participants with longer work year, hypertension and positive for blood lead. There was no statistically significant association between gender, age, smoking, drinking, whether wear mask or not and renal dysfunction (Table 2).

**Table 2:** Univariate analysis of the risk factors of renal dysfunction in paint workers

	Number of subjects	Number of renal dysfunction	Prevalence of renal dysfunction	χ2	*P* vaule
Age (years)				5.850	0.054
< 25	149	13	8.72		
25~	411	48	11.68		
37~ 59	187	32	17.11		
Gender				0.003	0.959
Male	706	88	12.46		
Female	41	5	12.20		
Work year (years)				12.146	0.002
< 2	195	13	6.67	Reference	
2~	381	48	12.60	4.793	0.029
9~ 32	171	32	18.71	12.261	0.000
Smoke				0.265	0.607
Yes	356	42	11.80		
No	391	51	13.04		
Drink				0.106	0.745
Yes	414	53	12.80		
No	333	40	12.01		
Wear mask				0.254	0.881
Never	41	6	14.36		
Occasionally	170	20	11.76		
Frequently	536	67	12.50		
Wash hands before meals				1.383	0.501
Never	50	7	14.00		
Occasionally	215	22	10.23		
Frequently	482	64	13.28		
Blood pressure				8.119	0.017
Low-normal blood pressure	509	56	11.00	Reference	
High-normal blood pressure	185	24	12.97	0.517	0.472
Hypertension	53	13	24.53	8.154	0.004
Blood lead				15.298	0.000
Negative	677	74	10.93		
Positive	70	19	27.14		

Because univariate analysis found the difference of prevalence between high-normal blood pressure group and low-normal blood pressure group was nonsignificant, those two group was merged into normal pressure group in multi-variable unconditional logistic regression analysis, all variables involved in logistic regression analysis were listed in Table 2, if *P* value ≤ 0.05, we defined it as positive, otherwise negative. The results of logistic regression analysis showed that renal dysfunction of paint workers was associated with longer working year (OR = 1.699, 95% CI: 1.226~ 2.355), blood lead positive (OR = 2.847, 95% CI: 1.577~ 5.139) and hypertension (OR = 2.192, 95% CI: 1.103~ 4.359) (Table 3).

**Table 3:** Risk factors of renal dysfunction and their interaction in low-level lead exposure paint workers

Variable	B	Wald test	P	OR	95% CI of OR
Work year (year)	0.530	10.143	0.001	1.699	1.226~ 2.355
Blood pressure (mmHg)	0.785	5.011	0.025	2.192	1.103~ 4.359
Blood lead (mg/l)	1.046	12.059	0.001	2.847	1.577~ 5.139
Constant	−4.308	69.828	0.000	0.013	

Of the major risk factors associated with renal dysfunction of paint workers, blood pressure and blood lead were controllable variables, so we further investigated the interactions between blood pressure and blood lead using stratified analysis, the results indicated that when blood pressure was normal, the association between low- level of lead exposure and renal dysfunction was statistically significant (OR = 2.784, 95% CI: 1.475~ 5.225). When blood lead was negative, the association between hypertension and renal dysfunction was statistically significant (OR = 2.181, 95% CI: 1.005~ 4.730). In cases where the hypertension and blood lead positive existed at the same time, the risk of kidney dysfunction was the largest (OR = 8.723, 95% CI: 2.131~ 35.710). RERI, API, and S were 4.758, 54.5% and 2.604 respectively, which indicated that there was a positive interaction between above two factors (Table 4).

**Table 4:** Analysis of interaction between blood pressure and blood lead on renal dysfunction in paint workers

Blood pressure	Blood lead	Number of renal dysfunction	Number of non-renal dysfunction	OR	95% CI of OR
Normal	negative	65	567	1	
Normal	positive	15	47	2.784	1.475~ 5.225
Abnormal	negative	36	9	2.181	1.005~ 4.730
Abnormal	positive	4	4	8.723	2.131~ 35.710
RERI			4.758		
API (%)			54.5		
S			2.604		

## Discussion

Urea nitrogen and creatinine are often used as indicators for renal function in clinical. However, these two indicators only become abnormal at the terminal stage of disease and are often subject to the diet and metabolism of the individuals, thus incapable of reflecting the renal dysfunction promptly and accurately. β2- MG is an alternative indicator for renal dysfunction. It is a small molecular globulin produced by lymphocyte, blood cells and polymorphonuclear leukocytes with a very constant synthetic ratio and burst size. 99.9% of β2- MG filtered by glomerulus could be absorbed by proximal renal tubules. Once the proximal renal tubules are lightly damaged, the amount of β2-MG in urine will soar [7]. Therefore, the amount of β2-MG can acutely reflect the damage of proximal renal tubules. Hence, it is of great significance in helping diagnosing renal dysfunction at its early stage [8]. According to our investigation, 12.37% of the study population suffered from renal dysfunction, which is much higher than some other participants. The investigation of Zhang LX [9] et al. showed that 10.8% of the study population of a national representative sample of 47,204 Chinese suffered from chronic renal dysfunction in 2009. And the prevalence of renal dysfunction of general population in UK and Canada were 5.85% [10] during 2007 to 2009 and 4.54% [11] in 2010, respectively. One hypothesis for the high percentage of renal dysfunction found in our study is the low level of lead exposure has increased the risk of renal dysfunction.

This investigation found that the longer painter work, the greater of the risk they have renal dysfunction. Working year, on one hand, had a positive correlation with age, which itself was a independent risk factor for renal dysfunction [12–14]; On the other hand, the longer one work, the longer one exposed to the lead. As a result, more leads accumulated in bodies would lead to high risk of renal dysfunction [15].

Our findings showed that hypertension is an independent risk of renal dysfunction. Studies of Bao YS [16] et al. and Bidani AK [17] et al. have shown similar results. One explanation was that changes of the hemodynamics in the kidney of the workers with hypertension lead to disturbance of the vascular activator such as reactive oxygen species and interleukin, which would cause the renal arterioles wall thickening and luminal stenosis, impose high pressure on glomerulus and increse filtering. Consequently, the amount of urine β2-MG go up accordingly [17].

In this investigation, 70 cases were detected positive for blood lead, ranging from 0.04 to 0.35 mg/l (mean 0.09 ± 0.06 mg/l), which are much lower than the limit of 0.40 mg/l stipulated by China and American etc. government for the occupations exposed to lead. Although the blood lead was at a very low level, those who tested blood lead positive were more likely to suffer from renal dysfunction than those tested negative.Some pertinent literature from other countries also found that low-level blood lead might lead to renal dysfunction [6, 15]. Muntner P [6] et al. found, from the NHANESIII (National Health And Nutrition Examination Survey III), that the prevalence of CKD was closely associated with the blood lead level in hypertension patients, with the blood lead level between 0.039~ 0.059 mg/l, the prevalence of CKD is 10.8%, and between 0.025~ 0.038 mg/l is 10.4%. The risk of CKD of these two groups are 1.69 and 1.56 times of those with blood lead <0.024 mg/l, respectively. Although CKD was not completely the same as renal dysfunction, both are associated with impaired renal function. Our study found that blood lead ≥ 0.04 mg/l and hypertension could increase the risk of renal dysfunction independently; moreover, the two factors also had obvious interaction on the renal dysfunction. When blood pressure is normal, the risk of renal dysfunction of those with blood lead positive (Pb-B ≥ 0.04 mg/l) is 2.784 times of those with blood lead negative (Pb-B < 0.04 mg/l). When blood lead level is negative, the risk of renal dysfunction of people with hypertension is 2.181 times of those with normal blood pressure. In cases the workers have both blood lead positive and hypertension, the risk of renal dysfunction was greatest (OR = 8.723), 54.5% of the results could be explained by the interaction between hypertension and blood lead. The possible mechanism of the interaction may included that: ① continuous hypertension causes glomeruli high pressure, and makes the glomerular filtration rate and urine β2- microglobulin increase; ② low level blood lead alone does damage to renal tubular epithelial cells, restrains ATP activity, and leads to tubular reabsorption dysfunction [18]. The co-existence of the two factors will promote renal dysfunction.

Two significant findings are achieved in this paper. One is that blood lead level much lower than the limit of 0.40 mg/l for the occupations exposed to lead may still increase the risk of renal dysfunction. Another is that there is a positive interaction to renal dysfunction between exposure to low level lead exposure and hypertension. From these findings, we can know that the limit for the occupational exposure to lead in China and other countries with same limit is not safety. It is also concluded that when utmost efforts are made to reduce occupational lead exposure, it is of great significance to control blood pressure of occupational population within a normal level to prevent renal dysfunction.

There is no denying that this research has some limitations. First, this is a cross- sectional survey, the lack of prospective follow-up limits the ability to make inferences regaring the causality of the associations. Second, although the utmost efforts were made to explore the influence factors of renal dysfunction, the factors which we analyzed still could not be ensured to be complete, there may exist other factors having something to do with renal dysfunction. Third, new reliable methods like ICP-MS available nowdays, but due to technical limitations, we used Graphite furnace atomic absorption spetrophotometry (WS/T20–1996) for the detection of lead, the accuracy maybe reduced. Finally, due to technical limitations, we didn’t measure other metals that might be associated with renal dysfunction such as Cd and Hg.

## Conclusions

Low-level lead exposure and hypertension not only have independent effect on renal dysfunction in paint workers, but also had obvious positive interaction in paint workers. Interventions aimed at blood lead and blood pressure at the same time will better prenvent from renal dysfunction.

## Additional file

Additional file 1: Questionnaire. In this file you can see the detail of questionnaire. (DOCX 17 kb)

## Abbreviations

API: Attributable proportions of interaction
CDC: Center for Diseases Control and Prevention
CI: Confidence interval
CKD: Chronic kidney disease
OR: Odds ratio
RERI: Relative excess risk of interaction
RR: Relative risk
S: the synergy index
SPSS: Statistical package for the social sciences
