# Supplementary material for: Risk factors of renal dysfunction and their interaction in level-low lead exposure paint workers
Source: BMC Public Health. 2018 Apr 20;18:526. doi: 10.1186/s12889-018-5475-9 (PMC5910569; doi:10.1186/s12889-018-5475-9)
Supplement: Supplementary file 1 — Questionnaire. In this file you can see the detail of questionnaire. (DOCX 17 kb) [file 12889_2018_5475_MOESM1_ESM.docx]

**Additional file 1**  **Questionnaire**

Name: No. :

1. General

(1) Gender:

□male

□female .

(2) Full Age: years

(3) Degree of Education:

□primary school and below

□secondary school,

□secondary vocational school or high school

□ junior college or above.

(4) Monthly household income per capita:

□under 1,000 yuan

□2000 yuan

□3,000 yuan

□more than 3,000 yuan.

2. Time:

Start painting work from (date) to (date), (working hours) every day.

3. Self-conscious symptoms, the following symptoms have occurred in the last 1 year (in the form of "√")

| Symptom | None | Occasionally | Often | Symptom | None | Occasionally | Often |
| --- | --- | --- | --- | --- | --- | --- | --- |
| Dizzy |  |  |  | Anorexia |  |  |  |
| Headache |  |  |  | Asthma |  |  |  |
| Insomnia |  |  |  | Nasal congestion |  |  |  |
| Dreaminess |  |  |  | Nasal cavity dryness |  |  |  |
| Hypomnesis |  |  |  | Chill |  |  |  |
| Fatigable |  |  |  | Hematuria |  |  |  |
| Anaesthesia |  |  |  | Constipation |  |  |  |
| tremor |  |  |  | Abdominal Distension |  |  |  |
| Bitter taste in mouth |  |  |  | Abdominal Pain |  |  |  |
| Dental ulcer |  |  |  | Hematochezia |  |  |  |
| Gomphiasis |  |  |  | Tinnitus |  |  |  |
| Phatnorrhagia |  |  |  | Lose hair |  |  |  |
| Joint sore |  |  |  | Hyposmia |  |  |  |
| Weakness of limbs |  |  |  | Nosebleed |  |  |  |

4. Do you smoke?

□No

□Yes, average per day, has been smoked for year (note: Not smoking for more than 1 year is quiting)

5. Do you drink alcohol?

□No,

□Yes, drinking white spirit liang / day, beer bottle/day,

drinking for year.

6. Personal hygiene

(1) Smoking, eating meals or other food in the workshop?

□Never

□Occasionally

□Often

(2)Do you wash your hands before meals?

□Never

□Occasionally

□Often

(3)Do you take a shower after work before going home?

□Never

□Occasionally

□Often

7. Personal protection

(1)Do you wear a mask at work?

□Never (turn to question 8)

□Occasionally

□Often

(2)How often does the mask change?

□~3 days

□~1 week

□~2 weeks

□ ~1 month.

(3)Do you wash your hands with banana water (thinner)?

□Never

□Occasionally

□Often

8.What is the reason for not wearing a mask?

□Troublesome

□Too dirty

□Useless

□Not received

□Other reasons

9. Regulatory knowledge

(1)Are you familiar the Law of Occupational Disease Prevention?

□No

□A little

□Full text reading

(2)Are you familiar the Labor Law?

□No

□A little

□Full text reading

10. Work environment

(1)Are toxic substances detected in the workplace?

□No

□I don't know

□Yes

(2)Are you satisfied with your current working environment?

□No

□It doesn't matter

□Yes.

(3)What are you not satisfied with? (Optional)

□Strong smell

□Large dust

□Loud sound

□Interpersonal tension

11. Are you satisfied with your current job?

□No

□Generally

□Yes

12. Your attitude in response to this questionnaire is:

□be serious

□be okey

□be casual

Survey date: (time)
